# Emotional tones in scientific writing: comparison of commercially funded studies and non-commercially funded orthopedic studies

**DOI:** 10.1080/17453674.2020.1853341

**Published:** 2020-12-02

**Authors:** Anath N V Steffens, David W G Langerhuizen, Job N Doornberg, David Ring, Stein J Janssen

**Affiliations:** a Department of Orthopaedic Surgery, Amsterdam, Amsterdam Movement Sciences (AMS), Amsterdam University Medical Centre, The Netherlands;; b Department of Orthopaedic & Trauma Surgery, Flinders University, Adelaide, Australia, Flinders Medical Centre;; c Department of Surgery and Perioperative Care, Dell Medical School, The University of Texas at Austin, Austin, TX, USA

## Abstract

Background and purpose — There is ongoing debate as to whether commercial funding influences reporting of medical studies. We asked: Is there a difference in reported tones between abstracts, introductions, and discussions of orthopedic journal studies that were commercially funded and those that were not commercially funded?

Methods — We conducted a systematic PubMed search to identify commercially funded studies published in 20 orthopedic journals between January 1, 2000 and December 1, 2019. We identified commercial funding of studies by including in our search the names of 10 medical device companies with the largest revenue in 2019. Commercial funding was designated when either the study or 1 or more of the authors received funding from a medical device company directly related to the content of the study. We matched 138 commercially funded articles 1 to 1 with 138 non-commercially funded articles with the same study design, published in the same journal, within a time range of 5 years. The IBM Watson Tone Analyzer was used to determine emotional tones (anger, fear, joy, and sadness) and language style (analytical, confident, and tentative).

Results — For abstract and introduction sections, we found no differences in reported tones between commercially funded and non-commercially funded studies. Fear tones (non-commercially funded studies 5.1%, commercially funded studies 0.7%, p = 0.04), and analytical tones (non-commercially funded studies 95%, commercially funded studies 88%, p = 0.03) were more common in discussions of studies that were not commercially funded.

Interpretation — Commercially funded studies have comparable tones to non-commercially funded studies in the abstract and introduction. In contrast, the discussion of non-commercially funded studies demonstrated more fear and analytical tones, suggesting them to be more tentative, accepting of uncertainty, and dispassionate. As text analysis tools become more sophisticated and mainstream, it might help to discern commercial bias in scientific reports.

There is ongoing debate as to whether commercial funding influences reporting of medical studies. While some studies of industry funding find differences in author conclusions, others do not (Clifford et al. 2002, Kjaergard and Als-Nielsen 2002). For instance, Lundh et al. (2018) included 75 papers that compared primary research studies sponsored by industry with studies with other sources of sponsorship. They found that industry sponsored studies present more favorable results (RR 1.3, 95% CI 1.2–1.4) and conclusions (RR 1.3, CI 1.2–1.5) as compared with studies that are not sponsored by industry. Conversely, Clifford et al. (2002) included 100 randomized controlled trials, of which 66% received funding, in whole or in part, from industry. They did not find a statistically significant association (p = 0.5) between funding source and trial outcome. However, this study may not be generalizable since it focused on recent publications of the top 5 general medical journals. Lower tier journals and specialty journals might not be as good at editing out bias. Furthermore, 100 studies might provide inadequate power for a small influence of funding.

Machine-learning-based tone analyzers are increasingly used to provide psycholinguistic analysis of text. For instance, the IBM Watson Tone Analyzer measures tones such as confidence and joy that might be more common in commercially funded studies if they are more promotional (Cloud 2019). Prior evidence suggests medical studies that use words such as “unique” and “novel” are more likely to be cited. Furthermore, there is evidence that men frame their studies more positively than women; women are more dispassionate in their writing (Lerchenmueller et al. 2019). As such, it is of interest to evaluate whether a tone analyzer could help identify a difference in reported tones between commercially and non-commercially funded studies.

This study addressed the primary null hypothesis that there is no difference in reported tones between abstracts of studies that were commercially funded and those that were not in 20 orthopedic journals, as analyzed by the IBM Watson Tone Analyzer. Secondarily, we addressed differences in tones in the introduction and discussion sections of the full paper.

## Methods

We conducted a systematic PubMed search to identify commercially funded studies published in 20 orthopedic journals between January 1, 2000 and December 1, 2019 (Appendix 1). The top 20 orthopedic journals were selected based on ranked impact factors according to Clarivate, a non-profit organization maintaining a website where journal statistics including impact factor are reported, on December 12, 2019. Commercial funding of studies was identified by including the names of 10 medical device companies with the largest revenue in 2019 in our search. We excluded letters to the editor, review articles, conference abstracts, animal and cadaveric studies, and studies not published in English.

A research fellow (ANVS) independently reviewed the conflict of interest (COI) statement for each study to confirm whether it was commercially funded or not. Commercial funding was designated when either the study or 1 or more of the authors received funding from a medical device company directly related to the content of the study. All COI statements were reviewed by a second research fellow (DWGL) to assess funding and confirm study eligibility. We excluded articles in which the COI statement did not mention funding, articles without a COI statement, and articles lacking an introduction or discussion.

For every selected commercially funded article, a similar article without commercial funding was matched 1 to 1 based on orthopedic journal, study design, and timeframe. A non-commercially—as indicated in the COI statement—funded study needed to have the same study design (e.g., randomized controlled trials, prospective cohort, retrospective cohort, case-series, case-control) as the commercially funded study, and to have been published within a time range of 5 years.

The PubMed search yielded 753 articles. We excluded 106 citations without a COI statement and 26 publications lacking an introduction or a discussion. Of the remaining 621 articles, we retained 138 commercially funded studies that could be matched with 138 non-commercially funded studies.

### IBM Watson Tone Analyzer

We used the IBM Watson Tone Analyzer to determine the reported tones of each article. The tone analyzer is based on the theory of psycholinguistics, wherein the relationship between behavior and psychological theories is explored. The IBM Watson Tone Analyzer is a machine-learning based model that has been trained on 96,000 customer-service Twitter conversations, rated by 5 annotators. According to IBM, the analyzer’s performance showed high accuracy against benchmark data. However, no reliability statistic or actual number has been reported to measure its performance (Cloud 2019). The tone analyzer reports emotional tones (anger, fear, joy, and sadness) as well as language style (analytical, confident, and tentative) (Table 1). In this study, every abstract, introduction, and discussion was copied separately into the tone analyzer. The reported scores vary between 0 and 1, in which < 0.5 means no tone, 0.5–0.75 means there is a tone detected, and > 0.75 means a strong tone is detected (Cloud 2019).

**Table 1.** Definition of dominant tones

Tones	Definition
Anger	Evoked due to injustice, conflict, humiliation, negligence, or betrayal. If anger is active, the individual attacks the target, verbally or physically. If anger is passive, the person silently sulks and feels tension and hostility
Fear	A response to impending danger. It is a survival mechanism that is a reaction to some negative stimulus. It may be a mild caution or an extreme phobia
Joy	Joy or happiness has shades of enjoyment, satisfaction, and pleasure. There is a sense of well-being, inner peace, safety, and contentment
Sadness	Indicates a feeling of loss and disadvantage. When a person can be observed to be quiet, less energetic, and withdrawn, it may be inferred that sadness exists
Analytical	A person’s reasoning and analytical attitude about things
Confident	A person’s degree of certainty
Tentative	A person’s degree of inhibition

From IBM Cloud Docs. Personality Insights. Available at: https://console.bluemix.net/docs/services/personality-insights/models.html#models. Accessed October 28, 2020.

### Statistics

The continuous data obtained by the IBM Watson Tone Analyzer was categorized into 2 groups: no tone (reported score < 0.5) and tone (reported score > 0.5). We used a McNemar test to compare dominant tones between commercially funded and non-commercially funded articles. A 2-tailed p-value less than 0.05 was considered statistically significant. All statistical analyses were performed using Stata® 15.0 (StataCorp LP, College Station, TX, USA).

To identify a difference with an effect size of 0.05 per dominant tone with sufficient power, we needed at least 84 papers per group, therefore 164 papers in total (α = 0.05, β = 0.10).

### Ethics, funding, and potential conflicts of interest

This study was exempt from institutional review board approval because it involves open-source data. We did not receive financial support for this study. All authors declare no conflicts of interest.

## Results

There were a similar number of tones that met the 0.5 threshold for commercially funded and non-commercially funded studies in the abstract and the introduction sections (Tables 2 and 3); for example, analytical tone was detected in 72% of study abstracts that were commercially funded, and 75% of study abstracts that were not commercially funded. In the introduction, analytical tone was detected in 84% of the studies that were commercially funded, and in 88% of the studies that were not commercially funded.

**Table 4.** Tone prevalence between non-commercially and commercially funded orthopedic journal articles — discussion

	Non-commercially funded article (n = 138)	Commercially funded article (n = 138)	
Tones	n (%)	n (%)	p-value
Anger	–	–	–
Fear	7 (5.1)	1 (0.7)	0.04
Joy	42 (31)	56 (41)	0.09
Sadness	67 (49)	73 (53)	0.5
Analytical	130 (95)	120 (88)	0.03
Confident	3 (2.2)	1 (0.7)	0.3
Tentative	51 (37)	49 (36)	0.8

– indicates no tones detected.

**Table 3.** Tone prevalence between non-commercially and commercially funded orthopedic journal articles ­— introduction

	Non-commercially funded article (n = 138)	Commercially funded article (n = 138)	
Tones	n (%)	n (%)	p-value
Anger	–	–	–
Fear	6 (4.4)	11 (8.0)	0.2
Joy	27 (20)	27 (20)	1.0
Sadness	43 (31)	37 (27)	0.4
Analytical	121 (88)	115 (84)	0.3
Confident	4 (2.9)	9 (6.6)	0.2
Tentative	43 (31)	30 (22)	0.09

– indicates no tones detected.

**Table 2.** Tone prevalence between non-commercially and commercially funded orthopedic journal articles — abstracts

	Non-commercially funded article (n = 138)	Commercially funded article (n = 138)	
Tones	n (%)	n (%)	p-value
Anger	1 (0.7)	1 (0.7)	1.0
Fear	9 (7.0)	8 (5.8)	0.8
Joy	43 (31)	40 (29)	0.9
Sadness	30 (22)	30 (22)	1.0
Analytical	104 (75)	100 (72)	0.6
Confident	20 (14)	18 (13)	0.7
Tentative	13 (9.4)	14 (10)	0.8

There was a difference in number of tones that met the 0.5 threshold in the discussion section (Table 4). Fear tones (non-commercially funded studies: 5.1%, commercially funded studies: 0.7%, p = 0.04) and analytical tones (non-commercially funded studies: 95%, commercially funded studies: 88%, p = 0.03) were more common in unfunded studies.

## Discussion

Tone analyzers may help determine whether there is bias in commercially funded studies. We found only limited difference in tone between commercially and non-commercially funded studies in orthopedic journals.

This study has several limitations. 1st, the relative infrequency of tones greater than 0.5 meant that we had to categorize tones as detected or not detected and could not analyze tone on its continuum. This might have introduced information bias. However, including only tones greater than 0.5 (leaving out studies in which no tone was detected) also leads to loss of information and therefore information bias. We felt that dichotomizing tone was the most adequate solution, as this method allowed for inclusion of all papers. 2nd, the IBM tone analyzer was trained on a large Twitter customer-support dataset. Although previously used in the context of medical studies, the reliability of the analyzer for medical journals is untested (Ottenhoff et al. 2018, Rajesh et al. 2018, Bakker et al. 2019, Karacic et al. 2019, Black et al. 2020, Langerhuizen et al. 2020). 3rd, we included only studies in high-impact orthopedic journals. This may potentially have introduced selection bias. However, we included studies from 20 different journals, and therefore consider this risk to be low.

The observation that the abstract and introduction were similar among commercially and non-commercially funded studies suggests that these sections of orthopedic studies are reported with comparable sentiment. The high percentage of abstracts and introductions that demonstrate analytical tone (72–88%) suggests that both may be reported in relatively dispassionate scientific language. This finding is in line with prior evidence demonstrating that there is no correlation between industry funding and more favorable reporting of a specific treatment in abstracts of orthopedic randomized controlled trials (Boutron et al. 2010, Arthur et al. 2020). In contrast, another study—evaluating randomized controlled trials in five orthopedic journals—demonstrated a substantially higher likelihood of presenting favorable outcomes in industry-funded studies (Khan et al. 2008). In addition, studies from ophthalmology and emergency medicine journals found that industry-funded trials were more likely to present the results as positive. In a study of topical prostaglandin research in ophthalmology, the conclusion presented in the abstract was not consistent with the statistical results in 18 of 29 of the commercially funded studies and none of the 10 non-commercial studies (Alasbali et al. 2009). In a review of emergency medicine clinical trial abstracts with no significant differences, a positive spin (i.e., selective reporting of significant differences, promotion of non-significant differences, favorable interpretation of non-significant results, or claimed benefit in spite of no significant difference) was present in 15 of 21 industry-funded trials compared with 35 of 93 non-industry funded trials (Reynolds-Vaughn et al. 2019). These are 2 of the many studies presenting evidence that industry-funded trials are associated with positive, proindustry study findings (Kjaergard and Als-Nielsen 2002, Bhandari et al. 2004, Alasbali et al. 2009, Boutron et al. 2010, Lundh et al. 2018, Lerchenmueller et al. 2019, Arthur et al. 2020). Among 186 registered randomized controlled trials comparing generic and brand-name drugs, only 46% were published within 4 years of completing the trial: 71% sponsored by a company with financial gains from both the generic and brand-name drugs, 28% comparing drugs from competing companies, and 46% with a non-profit sponsor (Flacco et al. 2016).

Our finding that fear and analytical tones were slightly more common in the discussion of non-commercially funded studies suggests the authors of non-commercially funded studies might be addressing uncertainty more directly, while also being less promotional and more dispassionate and analytical. Although we found a statistically significant difference, we consider this finding not to be a large clinically relevant difference.

Our finding that commercially funded studies have tones comparable to non-commercially funded studies in the abstract and introduction, but not in the discussion, suggests that abstracts and introductions might be more carefully edited to remove self-promotion than the discussion section of the paper. The discussion section of non-commercially funded studies has more fear and analytical tones, suggesting they might be more tentative, accepting of uncertainty, and dispassionate. As text analysis becomes more sophisticated, it might be able to discern commercial bias in scientific reports.
